# Efficacy and safety of the injection of the traditional Chinese medicine salviae miltiorrhizae and ligustrazine hydrochloride for the treatment of perioperative period of fracture: A meta-analysis of randomized controlled trials

**DOI:** 10.1097/MD.0000000000019777

**Published:** 2020-04-17

**Authors:** Jialong Xie, Shichun Chen, Shaobo Ding

**Affiliations:** Department of Pharmacy, Dongguan People's Hospital, Dongguan, Guangdong, China.

**Keywords:** fracture, ligustrazine hydrochloride, perioperative period, salviae miltiorrhizae, traditional Chinese medicine preparation

## Abstract

**Background:**

The injection of the traditional Chinese patent medicine salviae miltiorrhizae and ligustrazine hydrochloride injection (SMLHI) has been widely used in treatment of various diseases such as angina pectoris or ischemic stroke in China. We aim to evaluate the efficacy and safety of SMLHI for the treatment of perioperative period of fracture.

**Methods:**

A systematic literature search was performed in seven medical databases from their inception until February 2019. 16 studies with randomized controlled trials, totaling 1589 patients, were included in this meta-analysis. The included studies were assessed by the cochrane risk of bias and analyzed by Review Manager 5.3 software.

**Results:**

The meta-analysis showed that SMLHI for the treatment of perioperative period of fracture was significantly better compared with the control group in terms of the total effective rate. The result showed that SMLHI could significantly reduce the risk of deep vein thrombosis and inflammatory cytokines. Furthermore, the result showed that SMLHI could significantly improve the coagulation function indexes such as prothrombin time, plasma fibrinogen and D-Dimer (*P* < .0001).

**Conclusions:**

This meta-analysis demonstrated that SMLHI may be more effective and safe for the treatment of perioperative period of fracture. However, further and higher quality randomized controlled trials are required to prove treatment outcome.

## 1 Introduction

Fracture has accounted for more than 50% of all the injuries with the increasing aging of country and the increasing of traffic accidents and it is estimated an approximate of 8 million fractures in the United States every year.^[1]^ Deep vein thrombosis (DVT) is 1 of the most common complications in patients during perioperative period of fracture and DVT prophylaxis is essential after trauma, especially in patients with lower extremity or pelvic fractures.^[2,3]^ Previous studies have reported the incidence of DVT in perioperative period of fracture ranged from 8.2% to 61.3%.^[4]^ Antithrombotic therapy is currently used for the prevention of DVT and the study of Pelaez-Damy et al^[1]^ demonstrated the incidence of asymptomatic DVT after a major orthopedic surgery without prophylaxis reportedly ranges from 30% to 80%. The low molecular weight heparin (LMWH) and rivaroxaban are the most common antithrombotic drugs. But, the study^[5]^ suggested that prolonged use of LMWH may induce osteoporosis by modifying the bone metabolism and it was a risk factor causing delay in bone healing. The high cost of rivaroxaban also limits its wide application in antithrombotic therapy.^[3]^

Salviae miltiorrhizae and ligustrazine hydrochloride injection (SMLHI) is a phytochemical drug that is synthesized by tanshinol [chemical name: β-(3, 4-dihydroxyphenyl) lactic acid] and Ligustrazine Hydrochloride (chemical name: 2,3,5,6-tetramethyl pyrazine hydrochloride).^[6]^ Basic studies^[7]^ were performed that tanshinol, a component of SMLHI can increase coronary blood flow, improve microcirculation, reduce extent of myocardial ischemia, and protect the ischemic myocardium. Ligustrazine, the other component of SMLHI can inhibit platelet aggregation and fibrosis and regulate the lipid Metabolism. Modern research has shown that SMLHI can increase the blood flow of limbs and open collateral circulation, improve microcirculation. At the same time, it can improve blood rheology index and the coagulation function indexes. SMLHI has shown certain advantages for the treatment of perioperative period of fracture and has been widely used for more than 10 years in China. However, the use of SMLHI for the treatment of perioperative period of fracture in other countries is not an attractive medicine and the clinical efficacy of SMLHI combined with some western medicine was not certain. Therefore, our study included 16 randomized controlled trials (RCTs) with a total of 1589 patients who were included in order to acquire high-quality evidence for the clinical efficacy and safety of SMLHI for the treatment of perioperative period of fracture.

## 2 Methods

### 2.1 Literature search

We searched clinical studies databases, including PubMed, Embase, ClinicalTrials.gov, CBM, CNKI and Cochrane Central Register of Controlled Trials, from their inception until February 2018. We used the following search terms:

(1) “salviae miltiorrhizae and ligustrazine hydrochloride injection,” “danshenchuanxiongqin injection” connected with “OR,”
(2) “Fracture”, “Fractures, Bone”, “Fracture, Spiral”, “Broken Bones” connected with “OR,”
(3) “randomized controlled” or “clinical Trials”.

Then, the above search terms of (1), (2) and (3) were connected with “AND”. We manually searched the references of the original and review articles for possible related studies.

### 2.2 Study selection

For the systematic review, we searched 16 clinical studies following criteria:

(1) studies of patients undergoing major orthopedic surgery (femoral head replacement, total hip replacement or tibia and fibula surgery) were eligible.
(2) the control group received standard therapy (anti-infective therapy, complement of blood volume or functional exercise) or western medicine treatment LMWH or aspirin therapy.
(3) studies including patients who received SMLHI combined with western medicine treatment therapy in the experimental group.
(4) studies reported as RCTs,
(5) studies that reported efficacy and safety issues.

Exclusion criteria:

(1) We excluded studies that reported as non-RCTs, summary, case report and summary of the meeting.
(2) We excluded studies that reported outcomes as aggregate, without providing information on the components of the outcome.
(3) We screened the included studies that patients with family history of thrombosis, coagulation disorders or active bleeding, severe hepatic and renal insufficiency, Long-term use of anticoagulant or antiplatelet drugs (warfarin, aspirin or clopidogrel) were excluded.

### 2.3 Main outcome

The main outcome we extracted from RCTs were the total effective rate, DVT, Inflammatory cytokines and coagulation function.

### 2.4 Data extraction and quality assessment

Two of the authors independently extracted the data of the literature and carried out a quality assessment process according to the predefined inclusion criteria. Differences between the 2 authors were resolved by discussion with the third author. We used the cochrane risk of bias tool for the quality evaluation of the RCTs. This quality evaluating strategy included criteria concerning aspects of random sequence generation, allocation concealment, blinding of participants and personnel, blinding of outcome assessors, incomplete outcome data, selective reporting, and other bias.

### 2.5 Statistical analyses

In this meta-analysis, all statistical analyses were performed using RevMan software version 5.3 and we used odds ratio (OR) with 95% confidence interval (CI) for the analyses of dichotomous data. OR ratio is known as odds ratio, a common index in epidemiological study. It mainly refers to the ratio of the number of exposed people and non-exposed people in experimental group divided by the ratio of the number of exposed people and non-exposed people in the control group. CI is known as confidence interval, is used to estimate the range of parameters. The continuous data were presented as weighted mean difference (MD) or SWD with 95%CI. Heterogeneity between the studies was determined using the chi-square test, with the *I*^2^ statistic, where *I*^2^ < 25% represents mild inconsistency, values between 25% and 50% represent moderate inconsistency, and values >50% suggest severe heterogeneity between the studies. We defined *I*^2^ > 50% as an indicator of significant heterogeneity among the trials. We used random-effects models to estimate the pooled results to minimize the influence of potential clinical heterogeneity among the studies and the statistical significance was assumed at *P* < .05. Subgroup analyses were assessed using the *χ*^2^ test. Sensitivity analyses were performed to evaluate the robustness of merged results, by removing individual studies. Publication bias was assessed by means of funnel plots.

### 2.6 Ethics and dissemination

This meta-analysis is based on published research results, so we did not apply for ethical approval and patient consent. We will submit our meta-analysis which evaluates the Traditional Chinese Medicine Salviae Miltiorrhizae and Ligustrazine Hydrochloride for the Treatment of Perioperative Period of Fracture to a peer-reviewed journal for publication or conference presentations.

## 3 Results

### 3.1 Search results

**Figure 1:**
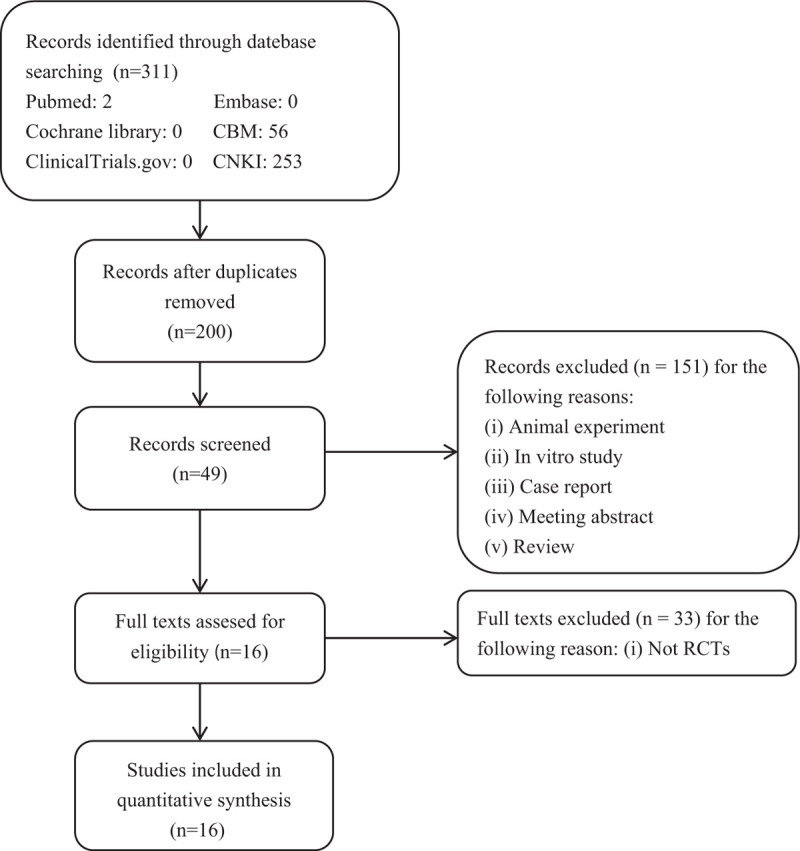
Flow chart and strategy of the meta-analysis.

A systematic search of studies published until February 2019 was performed through PubMed, Embase, ClinicalTrials.gov, CBM, CNKI and cochrane central register of controlled trials databases since their inception. A total of 311 literatures were searched and 16 studies were included in the inclusion criteria. The literature search procedure is shown in Figure 1.

### 3.2 Study characteristics

The included studies were 16 RCTs with a total of 1589 patients: the experimental group of SMLHI combined with LMWH and the control group with LMWH (8 studies). The dosage of SMLHI was almost 10 mL every day and the duration of both experimental and control group was about 10 to 14 days. More general characteristics of the included studies are listed in Table 1.

**Table 1:**
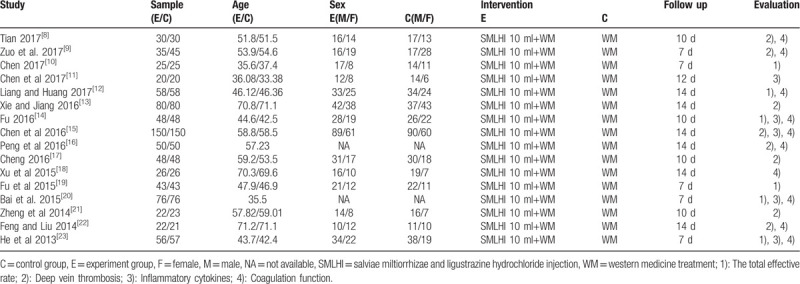
Characteristics of included articles.

### 3.3 Quality assessment

**Figure 2:**
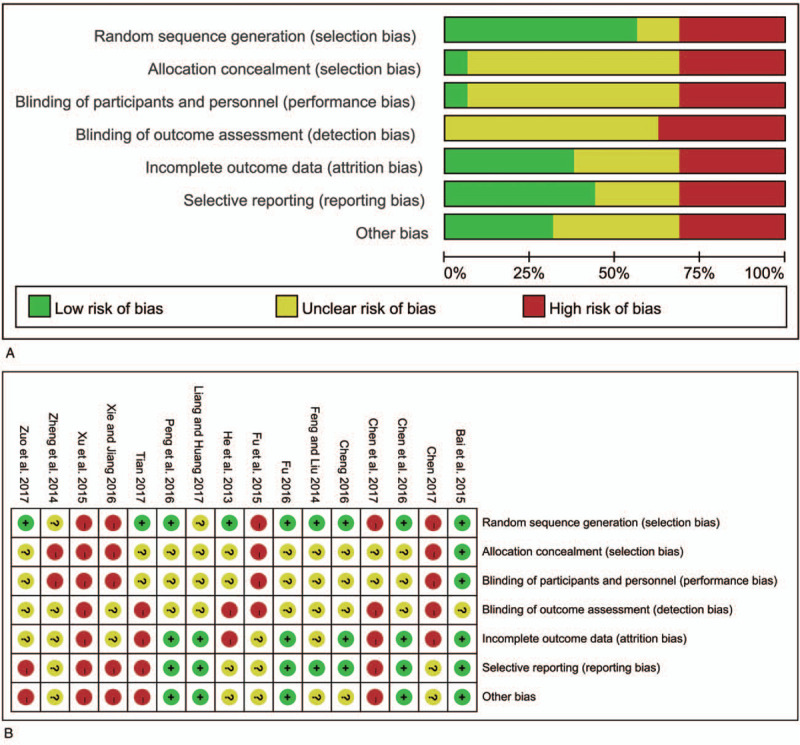
Quality of RCTs according to the Cochrane Collaboration Manual. (A: Summary of RCTs quality showing the percentage of RCTs satisfying each risk of bias graph. B: Detailed item-by-item analysis of the risk of bias summary.). RCT = randomized controlled trial.

The risks of bias in the included studies were evaluated by the Cochrane assessment tool and these results are summarized in Figure 2. Nine study was at low risk of bias for random sequence and reported the method of random allocation. Ten studies were at an unclear risk of bias for blinding of participants and personnel according to the Cochrane collaboration tool. Six studies reported methods with a low risk of attrition bias and 5 studies reported a low risk of reporting bias.

### 3.4 Major outcomes

#### 3.4.1 The total effective rate

**Figure 3:**
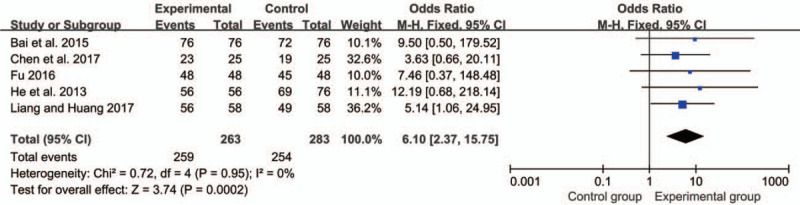
Forest plot of the meta-analysis with the total effective rate.

The total effective rate was reported in 5^[11,12,14,20,23]^ studies with a total of 263 patients treated with SMLHI and 283 patients in the control group. We used a fixed-effects model after the test for heterogeneity (*I*^*2*^ = 0 < 50%). The meta-analysis showed that SMLHI for the treatment of perioperative period of fracture was significantly better compared with the control group in terms of the total effective rate (OR = 6.10, 95%CI = 2.37, 15.75, *P* = .0002) (Fig. 3).

#### 3.4.2 DVT

**Figure 4:**
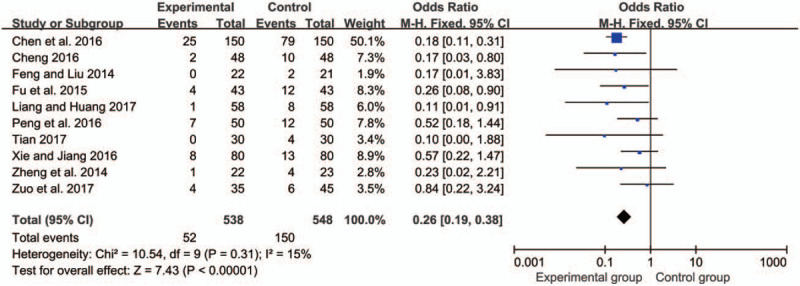
Forest plot of the meta-analysis with deep vein thrombosis.

There were 10^[8,9,12,13,15,16,17,19,21,22]^ studies with a total of 538 patients treated with SMLHI and 548 patients in the control group. After the test for heterogeneity (*I*^*2*^ = 15% < 50%), we used fixed-effects model. The result showed that SMLHI could significantly reduce the risk of DVT (OR = 0.26, 95%CI = 0.19, 0.38, *P* < .00001) (Fig. 4).

#### 3.4.3 Inflammatory cytokines

**Figure 5:**
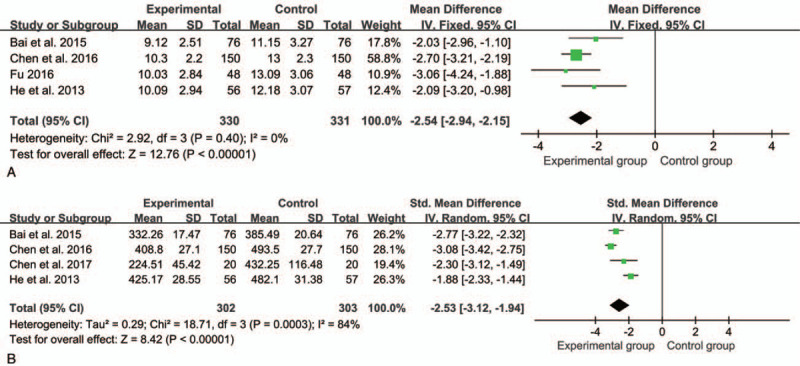
Forest plot of meta- analysis of inflammatory cytokines between experimental group and control group (A: Forest plot of C-reactive protein; B: Forest plot of tumor necrosis factor-α.).

There were 4^[14,15,20,23]^ studies with a total of 661 patients in regard to C-reactive protein and 4^[11,15,20,23]^ studies with a total of 605 patients in regard tumor necrosis factor-α. The result showed that SMLHI could significantly reduce the risk of inflammatory cytokines such as C-reactive protein (MD = -2.54, 95%CI = -2.94, -2.15, *P* < .00001) and tumor necrosis factor-α (standardized mean difference [SMD] = -2.53, 95%CI = -3.12, -1.94, *P* < .00001) compared with the control group (Fig. 5).

#### 3.4.4 Coagulation function

There were 10^[8,9,12,14,15,16,18,20,22,23]^ studies with a total of 1111 patients in regard to D-Dimer and 4^[12,14,15,18]^ studies with a total of 564 patients in regard to activation partial thromboplastin time and prothrombin time. The result showed that SMLHI could significantly improve the coagulation function indexes such as D-Dimer (SMD = -2.16, 95%CI = -3.13, -1.19, *P* < .0001), activation partial thromboplastin time (MD = 2.98, 95%CI = 0.64, 5.32, *P* = .01) and prothrombin time (MD = 0.79, 95%CI = 0.36, 1.23, *P* = .0003), compared with the control group (Table 2).

**Table 2:**
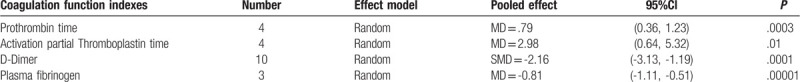
Results of meta-analysis of coagulation function indexes.

### 3.5 Subgroup analysis

#### 3.5.1 SMLHI + LMWH versus LMWH

Patients of perioperative period of fracture were treated with SMLHI and LMWH in experimental group and with LMWH in the control group. The results of subgroup analysis showed that SMLHI combined with LMWH therapy was more effective than LMWH in outcome of DVT. (OR = 0.33, 95%CI = 0.19, 0.58, *P* < .0001) and D-Dimer (SMD = -3.48, 95%CI = -5.68, -1.27, *P* = .002) (Table 3).

**Table 3:**
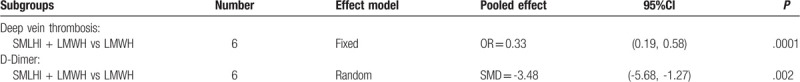
Subgroup analysis.

### 3.6 Heterogeneity and publication bias

**Figure 6:**
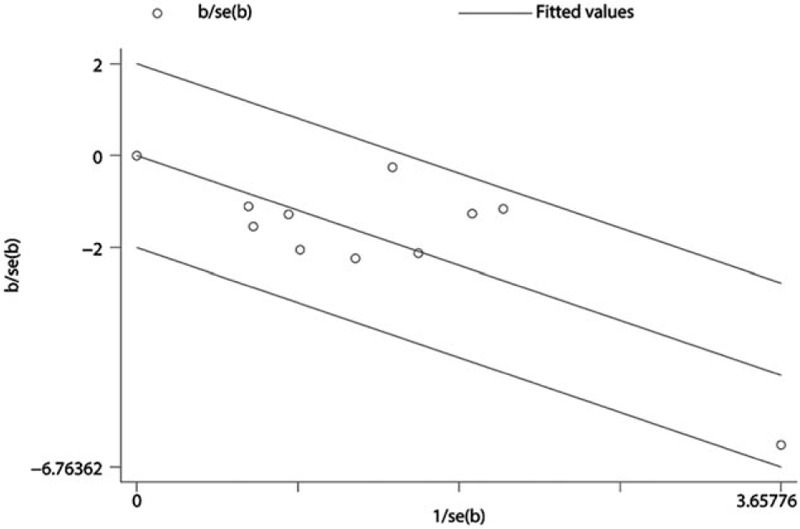
Meta-analysis of sensitivity with Galbraith plot of deep vein thrombosis.

**Figure 7:**
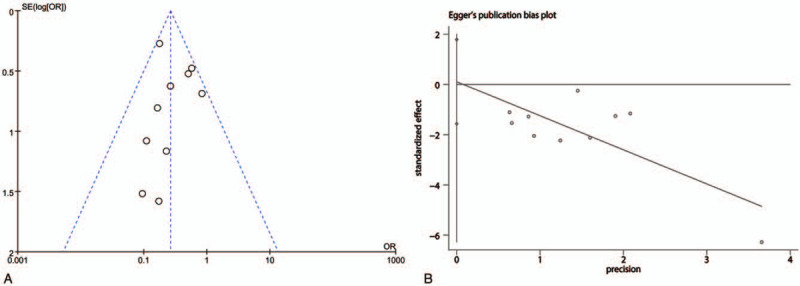
Meta-analysis of publication bias. (A: Funnel plot of deep vein thrombosis; B: Egger funnel plot of deep vein thrombosis.).

According to this meta-analysis, sensitivity analysis was performed using Galbraith plot of DVT. The results showed that there was no substantial change in DVT, indicating that the results of the meta-analysis were credible (Fig. 6). A significant symmetry was noted for distribution in funnel plots of DVT. The quantitation of Egger test with DVT (*P* > .889) indicated that publication bias was not obvious in the included studies (Fig. 7).

### 3.7 Safety

There were 4 studies that had reported the adverse reactions. The study^[10]^ of Chen found that there was 1 patient in the experimental group with venous pain and phlebitis during the intravenous injection. One study^[15]^ of Chen et al had reported that there was 1 patient with erythra in the experimental group and One study of Cheng^[17]^ had hematoma at the injection site. The study^[20]^ of Bai et al had nausea (3 case), vomiting (1case) and dizziness (1case) in the experimental group. No severe adverse drug reaction occurred in the experimental group and control group.

## 4 Discussion

### 4.1 Main outcome

Chinese patent medicine has been widely used in the treatment of various diseases such as cardiovascular diseases, sudden deafness, angina pectoris, or ischemic stroke. SMLHI as Chinese patent medicine can increase the blood flow of limbs and open collateral circulation, improve microcirculation. At the same time, it can improve blood rheology index and the coagulation function indexes. Our study included 16 RCTs with a total of 1589 patients who were included in order to acquire high-quality evidence for the clinical efficacy and safety of SMLHI therapy in perioperative period of fracture. The result showed that SMLHI for the treatment of perioperative period of fracture was approximately six times in the effect of total effective rate, compared with control groups. Many factors would lead to the formation of DVT in perioperative period of fracture:

(1) The patients can not be moved for long time during operation and the excessive pulling and rotation of extremities can also cause direct or indirect injury with peripheral vessels;
(2) The loss of blood after trauma and operation will cause the activation of coagulation function;
(3) the trauma, surgery, blood loss, anesthesia, limited movement during operation would lower blood pressure, slow flow of venous reflux.

Prevention of the formation of DVT has a vital role for patients during perioperative period of fracture. The study^[12]^ of Liang and Huang demonstrated the incidence of DVT in SMLHI therapy was significantly lower than that of the conventional group. The aggregated results of this meta-analysis also showed that SMLHI could significantly reduce the risk of DVT by 74%, compared with control groups.

The extent of inflammatory factors in the body of fracture patients is obvious, which can reflect the degree of stress in the body. The level of inflammatory factors in patients with fracture increased greatly, so the content of CRP and TNF-α can reflect the development and prognosis of patients with disease.^[14]^ The study^[11]^ of Chen et al indicated TNF-α levels of the SMLHI group were much lower after 12 days, compared with control groups. It is equally important to expression level and trend of IL-1 beta and IL-6 in serum is essential for clinical prediction of diagnosis of DVT. But there was no statistical difference between the control group and the experimental group.^[11]^ Whereupon,we aggregated results of this meta-analysis showed that SMLHI could significantly reduce the risk of inflammatory cytokines such as CRP and TNF-α, compared with the control group. However, there were not enough studies on the results of IL-1 beta and IL-6. We will include more studies to discuss the results in the future. D-Dimer is a clinical indicator to evaluate whether the formation of DVT and the coagulation function indexes of activation partial thromboplastin time, prothrombin time, plasma fibrinogen and thrombin time can accurately assess the blood clotting status of the body. The study^[18]^ of Xu showed the treatment group of SMLHI significantly improved plasma prothrombin time, activated partial thromboplastin time, plasma fibrinogen and D-Dimer. We aggregated results of this meta-analysis showed SMLHI can significantly improve the coagulation function indexes of D-Dimer, activation partial thromboplastin time and prothrombin time. Collectively, this meta-analysis demonstrated that SMLHI may be more effective for the treatment of perioperative period of fracture.

### 4.2 Subgroup analysis

The LMWH is the standard treatment and most common antithrombotic drugs.^[24]^ The study^[3]^ reported low-molecular-weight heparin had the prevention of venous thrombosis after internal fixation of hip fracture. In our meta-analysis of subgroup analysis, the results showed that SMLHI combined with LMWH therapy was more effective than LMWH in the outcome of DVT and D-Dimer. The results revealed that SMLHI is an excellent drug for the adjuvant therapies of perioperative period of fracture.

### 4.3 Safety

There were 4 studies that had reported the adverse reactions and no severe adverse drug reaction occurred in the experimental group and control group. A few patient had skin rash after the treatment of SMLHI and that consisted with the drug instructions. Tetramethylpyrazine is a compound with pyrimidine ring structure and strong antigenicity and hapten properties. This may be one of the reasons of adverse reactions caused by SMLHI. The another reason is that Chinese patent medicine contain many allergens, such as protein, tannin, pigment, resin, starch, volatile oil, mucus, and other macromolecules and if these substances enter blood, they maybe cause allergic reactions and lead to adverse reactions. It reminds doctor of the close observation of the patient's response to the drug during the infusion of SMLHI. If the patient had the headache, chills, fever, anaphylactic reaction and other symptoms, the doctor must immediately stop infusion. There was no serious adverse reaction in the incorporated literature and the results of this meta-analysis showed that SMLHI was safe for the treatment of perioperative period of fracture.

### 4.4 Limitations and critical considerations

We must be tapered in view of the limitations of this meta-analysis with low quality, high heterogeneity, and publication bias. The outcomes would lead to publication bias such as a lack of reporting about random sequence generation and concealment, especially in early and small trials. The review includes 16 RCTs, which were published in Chinese. Most of the studies were just referring to randomized trials, but there were no specific randomized trials of random sequence generation, allocation concealment, and blinding of outcome assessment. The methodological quality was generally low in most of the studies, which perhaps led to a risk of bias. Sensitivity analysis was performed using Galbraith plot and the results showed that there was no substantial change DVT and indicated that the results of the meta-analysis were credible. Symmetry was noted for distribution in funnel plots of DVT. The quantitative analysis of Egger test with DVT (*P* > .889) indicated that publication bias was not obvious in the included studies.

## 5 Conclusions

In summary, this systematic review and meta-analysis demonstrated that SMLHI may be effective and safe for the treatment of perioperative period of fracture. Subgroup analyses indicated that the clinical efficacy of SMLHI combined with western medicines of LMWH was significantly better than that of western medicine for the treatment of perioperative period of fracture. However, further and higher quality RCTs are required to prove its efficacy and provide meaningful evidence for clinical treatment due to the poor methodological quality and lack of adequate safety data.

## Author contributions

**Data Management:** Jialong Xie, Shichun Chen.

**Design and concept:** Jialong Xie, Shichun Chen, Shaobo Ding.

**Formal analysis and interpretation:** Shaobo Ding.

**Literature searching:** Jialong Xie, Shichun Chen.

**Methodology:** Jialong Xie.

**Software:** Shichun Chen.

**Supervision and Validation:** Shaobo Ding.

**Writing – original draft:** Jialong Xie, Shichun Chen.

**Writing – review and editing:** Shaobo Ding.
